# MSCs from polytrauma patients: preliminary comparative study with MSCs from elective-surgery patients

**DOI:** 10.1186/s13287-021-02500-9

**Published:** 2021-08-11

**Authors:** Raúl López, Gerardo J. Martí-Chillón, Juan F. Blanco, Carmen da Casa, Javier González-Robledo, David Pescador, Silvia Preciado, Sandra Muntión, Fermín Sánchez-Guijo

**Affiliations:** 1 Orthopaedic Surgery and Traumatology Department, University Hospital of Salamanca, Salamanca, Spain; 2 Haematology Department, University Hospital of Salamanca, Salamanca, Spain; 3 Instituto de Investigación Biomédica de Salamanca (IBSAL), Salamanca, Spain; 4 Universidad de Salamanca (USAL), Salamanca, Spain; 5 TerCel Network, ISCIII, Madrid, Spain; 6 Intensive Medicine Department, University Hospital of Salamanca, Salamanca, Spain; 7 Network Center in Regenerative Medicine and Cellular Therapy of Castilla y León, Salamanca, Spain

**Keywords:** Polytrauma, Bone regeneration, Mesenchymal stromal cells, Mesenchymal stem cells, MSC, Cellular therapy

## Abstract

**Background:**

Polytrauma is a major clinical problem due to its impact on morbidity and mortality, especially among the younger population. Its pathophysiology is not completely elucidated, and the study of the involvement of certain cell populations with therapeutic potential, such as mesenchymal stromal cells (MSCs), is an area of growing interest, as mesenchymal cells have anti-inflammatory, immunoregulatory, and osteogenic potential.

**Methods:**

In the present preliminary work, we have evaluated the characteristics of MSCs in terms of proliferation, immunophenotype, cell cycle, clonogenic capacity, and multilineage differentiation ability in a series of 18 patients with polytrauma and compared them to those from otherwise healthy patients undergoing elective spinal surgery.

**Results:**

MSCs from polytrauma patients displayed higher proliferative potential with significantly higher cumulative population doublings, increased expression of some important cell adhesion molecules (CD105, CD166), and an early pre-osteogenic differentiation ability compared to those of the control group.

**Conclusions:**

MSCs could potentially be of help in the repair process of polytrauma patients contribute to both cell-tissue repair and anti-inflammatory response. This potential should be further explored in larger studies.

**Supplementary Information:**

The online version contains supplementary material available at 10.1186/s13287-021-02500-9.

## Background

Polytrauma is a life-threatening clinical scenario where at least two different body regions are severely injured [1]. It is mainly originated by traffic accidents, falls, and interpersonal violence, and it is one of the main causes of disability and the first cause of mortality in people under 35 [2]. The pathophysiology is heterogeneous and could be determined by the trauma severity, number and type of organs affected, infections, treatments, and patient intrinsic factors [3]. Trauma severity evaluation could be assessed by diverse tools [4, 5], like the Injury Severity Score (ISS) [6], and the New Injury Severity Score (NISS) [7].

In the course of polytrauma, an inflammatory response to the injury may occur, the so-called systemic inflammatory response syndrome (SIRS) [8]. Critical polytraumatized patients usually develop SIRS after hemorrhage, hypoxia, and severe tissue injuries [3]. This inflammatory response can result in a poor evolution and lead to the development of a progressive multiorgan failure (MOF), even affecting organs not initially involved in trauma [9].

SIRS is triggered by the release of damage-associated molecular patterns (DAMPs) [10] which are recognized by immune cells, inducing a pro-inflammatory cascade signaling mediated by soluble factors release, like interleukins (IL) IL-1β, IL-6, or TNFα [11, 12]. Severe injuries stimulate the secretion of cytokines where their plasma concentration depends on the affected organs, the trauma severity, and the time course [13, 14].

As already indicated, the immune system is decisively involved. In this sense, the generated chemotactic gradient induced by DAMPs induces cell mobilization and recruitment. This includes mesenchymal stromal cells (MSCs), which can modulate the inflammatory microenvironment by some mechanisms, as paracrine and direct cell contact activity [15, 16]. MSCs are non-hematopoietic stem cell populations initially described in bone marrow (BM), but also present in adipose tissue, cord blood, and lungs, among other tissues, where they regulate the tissue microenvironment. MSCs have been also characterized to be involved in tissue restoration and regeneration after damage, like burns or trauma [17]. They are mobilized from BM and other niches to the injured tissues by different signaling pathways [18, 19], and their immunomodulatory activity is promoted by high levels of proinflammatory cytokines [1, 12]. Besides, MSCs promote the survival of damaged and apoptotic tissue cells [20, 21]. Despite the knowledge of these beneficial effects, the MSCs’ behavior would be directly related to the balance of all microenvironment signals generated after trauma.

Before the MSCs relocation, trauma molecular signals and cytokines can induce changes in MSCs phenotype, proliferative, multilineage, and their immunomodulatory potential, as seen after MSCs exposition to IFNγ, TNFα, and IL-1β [12, 22]. Based on these facts, some articles have described BM-derived MSCs behavior after trauma [18, 23, 24].

The presence of higher levels of proinflammatory cytokines and damage signals in plasma after trauma could pre-activate or condition the bone marrow MSCs’ activity in target tissues that may not occur in healthy patients. These differences may contribute to determining more precisely the MSCs’ inflammatory response and their implication in the outcome of polytrauma patients.

Thus, in the current work, we aimed to characterize MSCs obtained from BM of polytraumatized patients admitted to our Intensive Care Unit, and multiparametrically compare their proliferation, cell cycle, differentiation capabilities, and gene expression to that of otherwise healthy patients undergoing elective surgery.

## Methods

### Patients

For the above-mentioned purposes, we designed a prospective study including 18 polytraumatized Intensive Care Unit (ICU) patients and selecting as control group 25 patients undergoing elective spine surgery in our Institution. The study was conducted following the Declaration of Helsinki guidelines and local IRB (CEIm Area de Salud de Salamanca) approved the study (reference code 201912400). All participants (or their relatives in those who were unable to sign) signed a written informed consent form before their inclusion in the study.

Inclusion criteria included age > 18 years old, admission to ICU due to severe multiple trauma (polytrauma group, ISS > 16), or hospital-admitted for elective spine surgery (control group). We excluded patients showing any antecedent of malignancy, concomitant infectious disease, or immunosuppressive therapy.

Demographic data collected included patient’s age and gender, and polytraumatized ICU and post-ICU in-hospital length of stay (LOS). In addition, general biological values including hemogram, glycemic, ionogram, interleukins (IL), C-reactive protein, erythrocyte sedimentation rate (ESR), and procalcitonin were also recorded.

### MSCs isolation and characterization

In all cases, a 10 ml BM sample was obtained by aspiration from the iliac crest following standard procedures, which was subsequently transferred in sterile conditions to the cell culture facility of our Hospital, where the in vitro studies were performed. MSC isolation was performed as previously described [25]. Briefly, bone marrow mononuclear cells (MNCs) were obtained after Ficoll-Paque Plus (Sigma-Aldrich) gradient centrifugation. MNCs were cultured at a density of 1 × 10^6^ cells/cm^2^ in Dulbecco’s modified Eagle’s medium (DMEM—Gibco) supplemented with 10% of fetal bovine serum (Gibco) and 1% of penicillin/streptomycin solution (pen/strep—Gibco). Cells were grown in a humidified incubator (5% CO_2_) at 37 °C with a complete replacement of culture medium twice weekly discarding nonadherent cells. When culture confluence achieved 80–90%, cells were washed with Dulbecco’s phosphate-buffered saline (DPBS—Gibco) solution, trypsinized (Trypsin—Gibco), washed again, and counted with trypan blue (Sigma-Aldrich) using a hemocytometer. Harvested cells were replated in a new flask (this procedure is considered a passage) at a density of 5.000 cells/cm^2^. All experiments were performed within the six first passages.

### Immunophenotypic profile

After passage 3, harvested cells from the 10 samples of each group were washed with PBS and stained with monoclonal antibodies (McAb) for flow cytometry assay, which was performed according to MSC ISCT definition criteria [26]. The following McAb conjugated with either fluorescein isothiocyanate (FITC), phycoerythrin (PE), the tandem peridinin chlorophyll protein cyanine Cy5.5 (PerCP-Cy5.5), or allophycocyanin (APC) were used: CD34-FITC (Invitrogen); CD19-PerCP-Cy5.5, CD44-FITC, CD45-PerCP-Cy5.5, CD73-PE, CD90-FITC, CD166-PE, HLA-DR-PerCP-Cy5.5, (BD Biosystems); CD105-APC, CD106-FITC (R&D Systems); CD14-PE (Cytognos); and CD54-PE (Biolegend). 7-amino-actinomycin D (7AAD—Viability Stain solution, Biolegend) staining was employed to exclude dead cells (positive). Cells were acquired in a FACSCalibur flow cytometer (BD Biosystems) under a specific compensation and establishing an appropriate acquisition gate for forward- (FSC) and side scatters (SSC). Flow cytometry files were analyzed using Infinicyt^TM^ v.1.8 software (Cytognos). Both percentages of positive cells and Median Fluorescence Intensity (MFI) of each marker were measured and compared to unstained cells used as control.

### Clonogenic potential

To measure the clonogenic potential of stromal cells in each BM sample 1.5 × 10^5^, 7.5 × 10^5^, and 1 × 10^6^ MNCs were plated onto 25 cm^2^ culture flasks. Cells were cultured for 14 days in the same conditions described above with Iscove’s modified Dulbecco’s medium (IMDM—Gibco) supplemented with 10% FBS, 1% pen/strep, and 1% L-glutamine (Gibco). Fibroblast-like colony forming units (CFU-F) were counted after methanol fixation and May-Grünwald Giemsa conventional staining. A cell cluster containing more than 50 cells was considered a CFU-F colony. Ten control BM samples and ten polytrauma BM samples were employed for the CFU-F assay.

### Proliferation and cell cycle analysis

The proliferation capacity of MSCs was assessed in each passage with 10 samples from the control group and 9 from the polytrauma group measuring population doublings (PD). PDs were calculated using the formula indicated below, where ‘Np’ are the cells plated initially (4000 MSCs/cm^2^), ‘Nh’ the cells harvested. Cumulative population doublings (CPD) were also calculated as the summation of each PD value.

$$ \mathrm{PD}=\frac{\log\ (Nh)-\log (Np)}{\log 2} $$

For cell cycle analysis, MSCs (from nine control samples and ten polytrauma samples) were detached (trypsin), washed with PBS, and fixed with 70% ethanol. Subsequent steps were performed following Muse Cell Cycle Kit MCH100106 (Millipore-Merk) manufacturer’s instructions and reagents. MSC cell cycle distribution in G0/G1, S, and G2/M peaks was analyzed using the Muse v.1.5 software (Merck).

### Multilineage differentiation potential

Adipogenic differentiation of MSCs was determined (from eight control samples and six polytrauma samples) by culturing highly confluent 9.6 slide-flask (Nunc) in the adipocytic induction medium (MesenCult Adipogenic Differentiation, Stemcell), that was replaced twice per week. After 21 days of culture, cells were washed and fixed with paraformaldehyde. Then, slides were washed and stained with Oil-Red-O (Merck) for 30 min. Cell morphology changes and the number of adipocytes per field were measured in three different fields.

For chondrogenic differentiation, MSCs were cultured for 21 days with a specific medium (StemMACS ChondroDiff, MACS), and then assessed by the expression of specific genes, as indicated in the next section.

Osteogenic differentiation potential was evaluated by culturing MSCs at 3 × 10^5^ cells in a 9.6 cm^2^ slide-flask and grow up in an osteogenic medium (StemMACS OsteoDiff Media, MACS). The medium was replaced every 3–4 days for 10 days. After washing with PBS, cells were fixed with methanol for 5 min and washed again with PBS. Phosphatase alkaline activity was performed by incubating fixed cells with a solution of 5-Bromo-4-Chlore-3-indolyl phosphate (BCIP—Sigma-Aldrich) and nitro blue tetrazolium (NBT—Sigma-Aldrich) during 15–30 min. Cell morphology and mineral deposits were measured and compared to non-induced cells (cultured with complete DMEM). In order to quantify the mineralization potential of MSCs, cells were cultured in the same conditions in 6-well plates. Seven samples from the control group and five from the polytrauma group were employed for this purpose. After 21 days of culture, alizarin red staining was performed as described by Gregory et al [27]. Briefly, after fixation with methanol and washing twice with dH_2_O, 1 ml of alizarin red solution (Sigma) (pH 4.1) was added. After 20 min of agitation, two washing steps were performed and 800 μl of acetic acid was added while scrapping the flask surface. The slurry was then transferred to an Eppendorf tube which was heated at 85 °C for 10 min and cooled 5 min in ice. After centrifuging 16,000*g* for 27 min, the supernatant was neutralized with 200 μl of ammonium hydroxide. Then the final solution concentration was measure per triplicate in a 96-well transparent flat plate using a spectrophotometer (reading at 405 nm). The values obtained were normalized (with non-induced control cells from each sample) and used to calculate the mineralization product concentration following Lambert-Beer’s law.

### Genetic profile of MSCs

Total RNA was extracted from non-induced MSCs and differentiated MSCs in all differentiation mentioned conditions from at least 5 samples from each group. After 10 and 21 days of culture for osteogenic differentiation, and 21 days for adipogenic and chondrogenic, cells were trypsinized and washed with PBS. Then 1 ml of TRIzol (Merk) was added to all recovered cells. Following the TriPure RNA isolation protocol, a density gradient was established after adding 200 μl of chloroform and centrifuging for 30 min at 10,000 rpm. After removing the aqueous phase, RNA was precipitated with isopropanol and washed twice with 70% ethanol, and finally resuspended with dH_2_O.

The quality and quantity of RNA obtained were measured with NanoDrop (Thermo Fisher Scientific). A reverse transcriptase-polymerase chain reaction (RT-PCR—High-Capacity cDNA Reverse Transcription kit, Applied Biosystems) was performed before the quantification step. Real-time PCR (qPCR – TaqMan Fast Universal PCR, Applied Biosystems) was employed for gene expression quantification. Gene expression was measured using primers for the specific genes mentioned in Table 1. GAPDH was used as a housekeeping gene to normalize the expression among samples. All primers were acquired from Applied Biosystems. The relative quantification was calculated as 2^-ΔΔCt^.

**Table 1:** Primers employed for gene expression analysis

Function	Gene		PRIMER ID
Housekeeping gene	GAPDH	Glyceraldehyde-3-phosphate dehydrogenase	Hs99999905
Adipogenic differentiation	CEBPα	CCAAT enhancer binding protein alpha	Hs00269972
Adipogenic differentiation	PPARγ	Peroxisome proliferator-activated receptor gamma	Hs01115512
Chondrogenic differentiation	SOX9	SRY-Box 9	Hs00165814
Chondrogenic differentiation	COL1A1	Collagen type I alpha 1 chain	Hs00164004
Osteogenic differentiation	ALPl	Alkaline phosphatase	Hs00758162
Osteogenic differentiation	RUNX2	Runt-related transcription factor	Hs00231692
Osteogenic differentiation	DKK1	Dickkopf WNT signaling pathway inhibitor 1	Hs00183740
Osteogenic differentiation	SPARC	Osteonectin	Hs00277762
Osteogenic differentiation	SPP1	Osteopontin	Hs00959010
Immunomodulation	TGF-β	Transforming growth factor beta 1	Hs00998133
Immunomodulation	TNFα	Tumor necrosis factor alfa	Hs00174128
Immunomodulation	PTGS2	Prostaglandin-endoperoxide synthase 2	Hs00153133
Immunomodulation	IL-6	Interleukin-6	Hs99999032
Immunomodulation	IL-10	Interleukin-10	Hs00174086
Immunomodulation	IDO	Indoleamine 2,3-dioxygenase 1	Hs00984148
Other	BDH2	3-Hydroxybutyrate dehydrogenase 2	Hs00560373

### Statistical analysis

Statistical analysis was performed using IBM SPSS v.26 software. Continuous variables were expressed by mean and standard deviation, and median and interquartile range [Q1-Q3]. Normal distribution was assessed by Shapiro-Wilk tests, Q-Q plots and Kolmogorov Smirnoff Lilliefors corrected test. Related samples were compared by paired *T* test or Friedman test, as appropriate. Group comparison was performed by *T* Student’s test or *U* Mann-Whitney tests, as appropriate. Continuous correlations were assessed by linear regression models. Qualitative variables were expressed by percentages. Group comparisons were addressed by the chi-square test or Fisher’s Exact test, as appropriate. In all cases, *p* < 0.05 was considered statistically significant.

Figure artwork was performed using IBM SPSS, GraphPad Prism, and Microsoft Office 2016 software.

## Results

### Polytrauma patients’ hospitalization data

A total of 18 polytraumatized patients were enrolled in the study. Men represented 72.2%, and the mean age of the population was 51.56 ± 17.3 years. They showed a mean ISS of 25.22 ± 6.0 and NISS of 28.67 ± 9.0. The mean noted Glasgow scale on hospital admission was 10.89 ± 4.5. The Intensive Care Unit length of stay was 13.94 ± 11.3 days, leading to a whole in-hospitalization length of stay of 23.33 ± 14.9 days. The complete lab clinical values for polytraumatized patients upon hospital admission are summarized in Suppl. Table 1. During the in-hospitalization, we monitored hemoglobin, leukocytosis, and serum levels of C-reactive protein, lactate, and procalcitonin (Suppl. Figure 1). No statistically significant variance was observed in these monitored parameters (*p* > 0.05, in all cases). During the hospital stay, none of the participating polytrauma patients developed sepsis and/or MOF. Only one patient aged 85 died before hospital discharge.

A significant linear correlation between the severity of the trauma and the recovery days of the patients was observed (*p* < 0.001) (Suppl. Figure 1). The greater ISS, the more time was required for polytraumatized patients to be discharged (*B* = 0.283 [0.133–0.433], *p* = 0.001) (Suppl. Figure 1A), and ICU output (*B* = 0.397 [0.213–0.582], *p* < 0.001) (Suppl. Figure 1B). NISS showed an even greater correlation with ICU length of stay (*B* = 0.715[0.526–0.904], *p* < 0.001) (Suppl. Figure 1C). Concerning the lab clinical variables studied of polytrauma patients, TNFα levels also correlated to the number and severity of lesions measured by NISS (*B* = 0.930 [0.189–0.1670], *p* = 0.019) (Suppl. Figure 3).

### MSC culture and immunophenotypic characterization

Evaluable MSCs samples were obtained from 14 polytraumatized patients (71.4% men, median age 59 [33–66] years, mean age 52.9 ± 19.1 years), and 19 control patients (63.2% men, median age 60 [53–75], mean age 62.2 ± 11.3 years) that were further analyzed. No significant differences in biodemographic variables were noted (*p* > 0.05, in all cases).

**Fig. 1:**
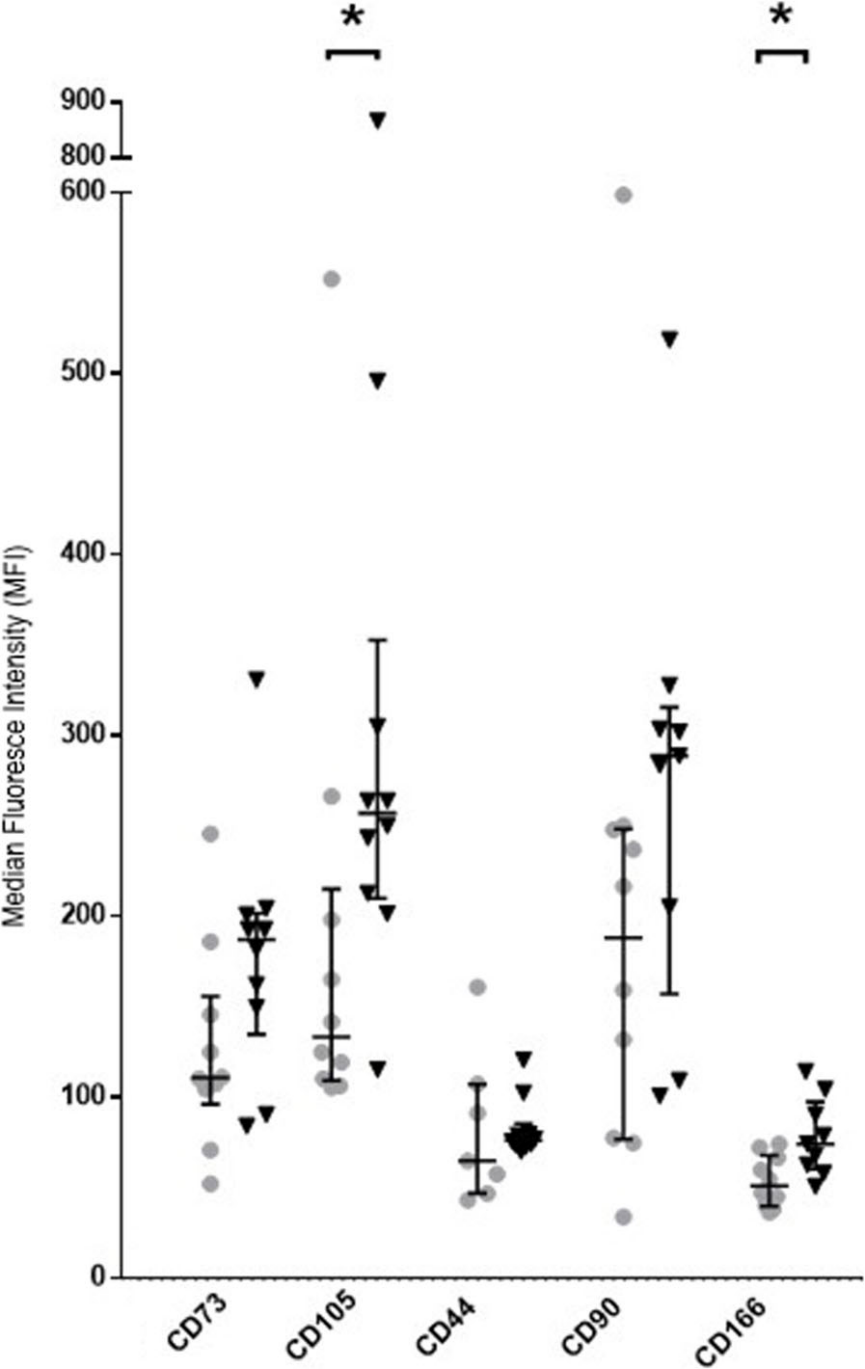
Mean fluorescence intensity (MFI) differential expression of positive MSC membrane markers. Expression of CD44, CD105, CD90, CD73, and CD166 in polytrauma MSCs (gray dots) and control MSCs (black triangles). Bars are expressing median and interquartile range. “*” indicates *p* < 0.05 on group comparisons

MSCs immunophenotyping is summarized in Suppl. Figure 4 MSCs samples showed similar viability (over 85%), and no significant differences (*p* > 0.05) were detected in membrane marker expression between both groups, and in all cases, MSCs fulfilled ISCT immunophenotypic definition criteria. However, we observed higher MFI values for CD105 (1.74 mean fold increase, *p* = 0.035) and CD166 (1.52 mean fold increase, *p* = 0.014) in MSCs from polytraumatized patients (Fig. 1).

### Proliferation and cell cycle analysis

**Fig. 2:**
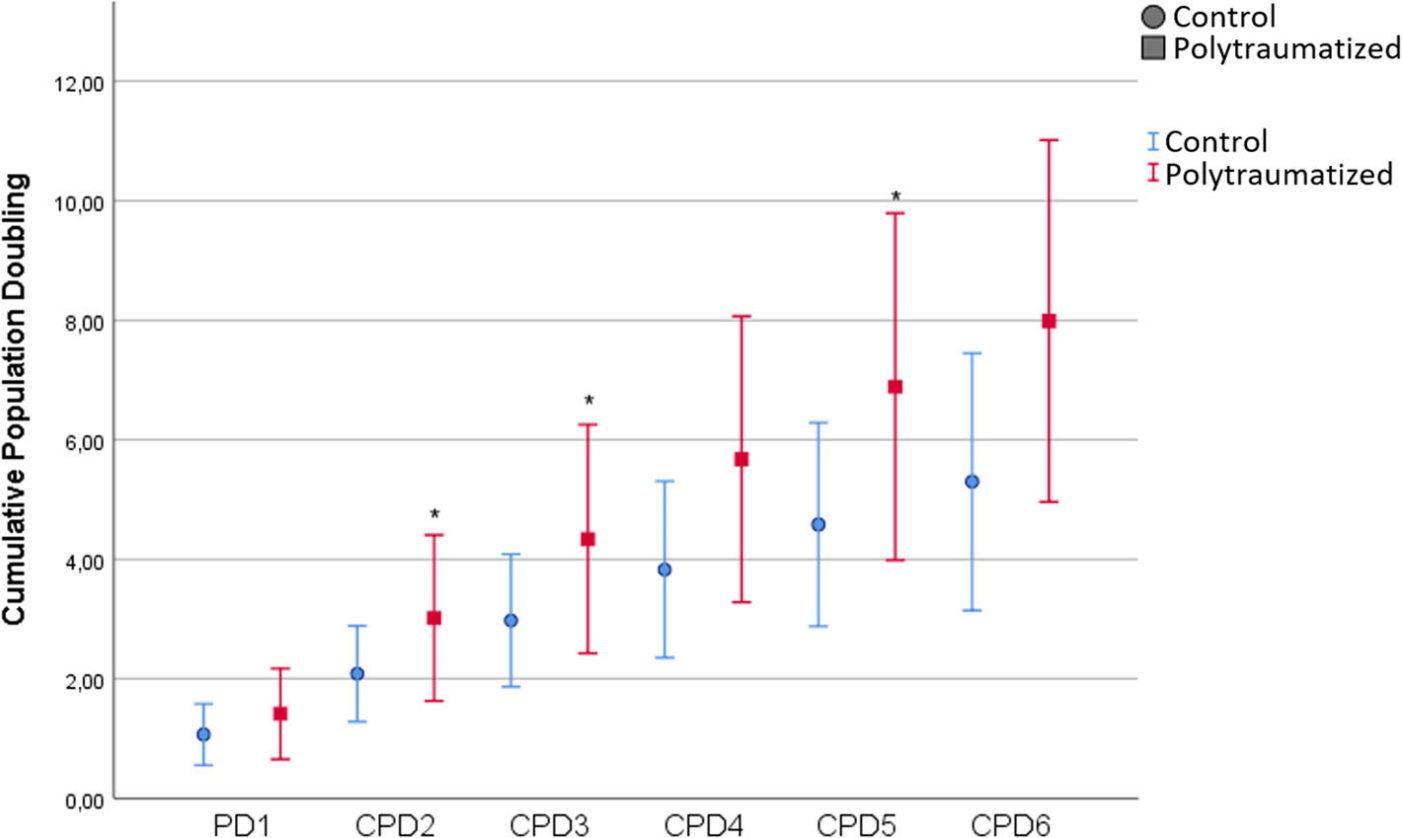
Mean cumulative population doubling (CPD) of control MSCs (blue) and polytraumatized-derived MSCs (red). Error bars are representing standard deviation. “*” indicates *p* < 0.05 on group comparisons at each stage

Regarding cell proliferation analysis, MSCs from polytrauma patients showed a significantly higher cumulative population doubling (Fig. 2). Although there were no differences between groups in the CFU-F clonogenic ability, its numbers in polytraumatized patients correlated with NISS (*B* = 0.746 [0.102–1.389], *p* = 0.027), and TNFα serum levels (*B* = 0.930 [0.247–1.614], *p* = 0.014).

Cell cycle analysis showed that polytraumatized MSCs seem to have a slightly higher subset in S (8.5% ± 1.1%) and G2/M (9.6% ± 5.0%) phases, and a lower subset in G0/G1 (83.8% ± 3.9%) phase, compared to control MSCs (phase S: 6.9% ± 0.6%, phase G2/M: 7.7% ± 3.6%, phase G0/G1: 79.8% ± 8.0%), although no statistical significance was observed (*p* > 0.05, in all cases).

### Adipocytic, chondrogenic, and osteogenic differentiation

Both groups of MSCs cells showed differentiation capacity into adipocyte, chondrocyte, and osteoblast after appropriate differentiation cultures.

**Fig. 3:**
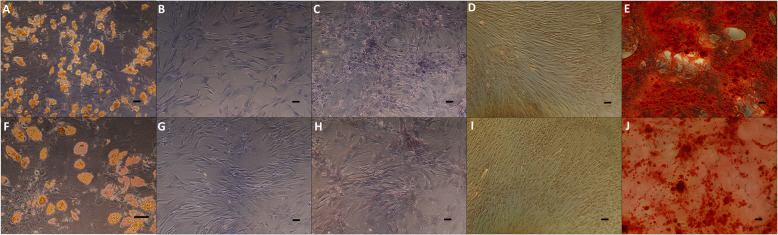
In vitro MSC multilineage differentiation. Control group (**A**–**E**) and polytrauma group (**F**–**J**). Oil-Red staining (10×) after 21 days of culture (**A**/**F**). Alkaline phosphatase staining (10×) after MSC culture with baseline expansion medium (**B**/**G**) and osteodiff medium (**C**/**H**) for 10 days. Alizarin-Red staining (10×) with baseline expansion medium (**D**/**I**) and osteodiff medium (**E**/**J**) for 21 days. Scale bar: 100 μm

**Fig. 4:**
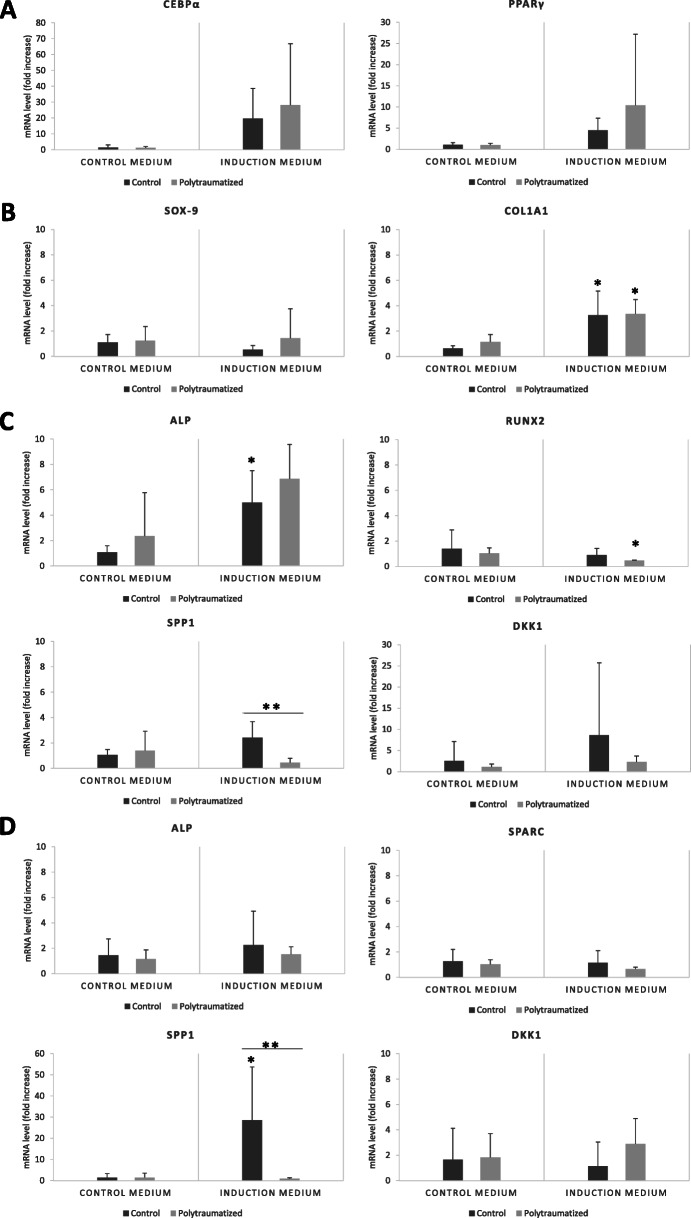
Fold increase in mRNA levels of the cited genes. For adipocyte-differentiated MSCs for 21 days (**A**), chondrocyte-differentiated MSCs for 21 days (**B**), and osteoblast-differentiated MSCs for 10 days (**C**) and 21 days (**D**)

There were no significant differences in mean adipocyte cell count per culture between groups (Fig. 3A/F). In addition, there was no difference in CEBPα and PPARγ expression between polytrauma and elective surgery patients after differentiation (Fig. 4a).

Regarding chondrogenic differentiation, both control and polytraumatized MSCs showed similar basal and induced SOX-9 and COL1A1 expression levels, as shown in Fig. 4b.

Finally, osteogenic differentiation capability of MSCs cultures on induction/basal media for 10 days and 21 days is shown in Figs. 3B/E, G/I and 4C/D.

Control and polytraumatized MSCs showed similar basal and induced ALP, DKK1, RUNX2, and SPARC expression levels at 10-days culture (*p* > 0.05, in all cases). However, SPP1 expression levels were statistically significantly lower in polytraumatized MSCs after 10 days of osteoblast differentiation compared to control MSCs (*p* = 0.009).

Regarding the mRNA expression analysis at 21-days osteoblast differentiation induction (Fig. 4d), we noted that SPP1 expression levels were again significantly lower polytraumatized MSCs (*p* = 0.008). Mineralization quantification at 21 days measured by alizarin red was similar baseline and after osteogenic induction in both experimental groups.

### Immunomodulatory genetic profile

Expression of pro-inflammatory, anti-inflammatory, and regulatory genes (TNFα, IL-6, TGFβ, IL-10, PTGS2, IDO, and BDH2) was analyzed (Suppl. Figure 5). There were no differences in gene expression after comparing MSCs from polytrauma with those from elective surgery patients (*p* > 0.05, in all cases).

## Discussion

The present work is among the scarce number of studies assessing the characteristics of BM-MSCs from polytrauma patients, which interest has grown in the last few years. One of the originalities of our study is the selection of the control group. While most studies lack a control group of cells is compared to those of healthy BM donors, we have selected a group of otherwise healthy patients undergoing elective spine surgery, since the single injury induced by the surgical process may elicit some of the stimuli that may modify MSCs properties and function. Besides, we have multiparametrically compared both groups in terms of cell expansion, proliferation, clonogenic ability, immunophenotype, genetic profile, and differentiation. Although the information on preclinical models of polytrauma is extensive, information on human samples is limited. In one study, MSCs from patients with unilateral trauma or osteoarthritis, but not with polytrauma, did not show any difference in proliferative and differentiation capacities comparing to non-traumatic and chronic bone disorder [28]. Nevertheless, in another study, when MSCs were stimulated in vitro with serum from polytrauma patients (right after hospital admission) revealed a significant increase in proliferative potential [12].

In our preliminary study, we have observed a higher proliferation ability of polytrauma MSCs, with similar high differentiation capability to elective-surgery MSCs (acting as the control group). This may be attributed to the hyperinflammatory status of these patients. Previous works have demonstrated the involvement of MSC in the inflammatory response and their immunomodulatory role has been established in some clinical scenarios [29–33]. These effects are related to several mechanisms, such as apoptosis, cell migration or recruitment, chemotaxis, killer cells’ inhibition [34]. Therefore, the presence of higher levels of proinflammatory cytokines and damage signals in plasma after trauma could condition BM-MSCs activity. We have observed a NISS correlation to TNFα polytrauma patients’ serum levels and CFU-F cell clonogenic ability, suggesting that inflammatory cytokines released after trauma may induce a higher MSCs proliferation capability. As previously described, TNFα promoted proliferation by increasing cell number/colony in synovial MSCs [35]. Polytraumatized-derived MSCs had greater proliferative potential than the control group showing higher cumulative growth throughout passages. This may be related to the intensity of the systemic inflammatory response [23, 35]. Polytrauma MSCs’ proliferation rate increment could be understood as a protective response, given the fact that MSCs not only may act as a modulator of the acute inflammatory environment but also promoting tissue regeneration (angiogenesis, cell survival, restoration of microenvironment homeostasis).

A direct correlation between the proliferation capacity and the CFU-F efficiency has been described [36]. Although we did not detect significant differences on CFU-Fs between polytraumatized and control patients, former work on polytrauma MSCs already showed a higher clonogenic capability of polytrauma MSCs compared to control, monofracture, and atrophic nonunion patients [23]. However, male MSCs from polytraumatized samples did not differ from those from the control group, whereas female MSCs showed a higher number of colonies than both male groups (control and polytraumatized) [23]. In our study up to 70% of polytraumatized patients enrolled were males, a condition that may reduce overall colony numbers. CFU-F values depend on intrinsic factors such as age, sex, pathology, or even sample quality (% MSCs/ml BM) [23, 37].

Regarding immunophenotype, although in all cases MSCs fulfilled ISCT definition criteria, polytraumatized-derived MSCs showed higher expression of CD105 and CD166 membrane markers in terms of MFI values. Endoglin (CD105) is a classical protein for MSCs identification [26]. Endoglin is also expressed in endothelial cells and participates in the TGFβ signaling pathway and cell adhesion, but its function in MSCs is still not fully determined. TGFβ levels increase in polytraumatized patients’ plasma [38]. After studying the MSCs immunomodulatory genetic profile, all samples expressed high levels of TGFβ, but without differences between groups. TGFβ is a cytokine involved in intercellular communications promoting cell proliferation, angiogenesis, adhesion, and migratory events that play a crucial role in further downregulation of inflammatory cytokines and MSC proliferation and differentiation. In murine adipose tissue-derived MSCs, differential expression of CD105 was related to a variation of MSCs differentiation and immunoregulatory capacity. CD105^-^ population showed higher differentiation potential. Similar MSCs growth kinetics, CFU-F potential, and MSCs markers were showed among positive and negative populations [39]. CD166 (ALCAM) is expressed in some progenitor cells, and their expression has been associated with cell adhesion and migration [40]. MSCs exposed to some major cytokines and anaphylatoxins from the serum of polytrauma patients upregulate genes involved in mobilization and homing [18]. These proteins allow MSCs to adhere, detach and migrate through the tissue matrix under chemoattractant stimuli. Furthermore, the endothelial barrier plays a crucial role in MSCs’ systemic distribution under inflammatory conditions by expressing adhesive interactions (diapedesis, transendothelial migration). MSCs preferentially transmigrate through TNFα activated endothelial cells into the inflammatory focus [41, 42]. Higher expression of these markers may suggest a higher MSCs migratory ability and could be induced as a response to the polytraumatic event since it has been well established the mobilization and migration of MSCs to tissue pro-inflammatory signals [18].

MSCs obtained from both experimental groups were able to differentiate into adipocytes, chondrocytes, and osteocytes under standard differentiation cultures, and no relevant differences were observed in both adipogenic and chondrogenic gene expression between them. Nevertheless, polytraumatized MSCs presented higher early osteogenic differentiation with higher levels of alkaline phosphatase and lesser DKK1 than the control group. Late differentiation hallmarks as SPP1 were reduced. This information has not been reported to date and is of potential biological interest, since an early osteogenic predisposition in polytraumatized patients, together with its anti-inflammatory ability may favor bone regeneration.

In inflammatory conditions, MSCs tend to produce anti-inflammatory molecules to modulate microenvironment and immune system dysregulation, as occurs after polytrauma [24]. In our study, the immunomodulatory profile of MSCs showed no differences in the expression of genes involved in immunomodulation between groups, which can be potentially due to the absence of continuous stimuli during culture expansion may reduce MSCs anti-inflammatory expression profile [12, 28]. For future studies, it would be interesting to evaluate the genetic profile of sorted non-expanded BM-MSCs during the first days after patient hospital admission.

Finally, our study is not exempt from some inherent limitations, since it is a preliminary study, in which the sample size is limited. Moreover, during the in-hospitalization process of polytraumatized patients, none of them developed MOF, so-hence MSCs characterization of polytraumatized patients with a worse SIRS developing into sepsis or MOF could not be performed, and our sample may not be fully representative of this heterogeneous entity.

## Conclusions

In summary, our preliminary study shows that MSCs from polytraumatized patients showed higher proliferative potential, increased cell adhesion molecules, and early pre-osteogenic differentiation ability compared to those of otherwise healthy patients undergoing elective surgery. They could potentially be of help in the repair process of these patients since MSCs from polytrauma patients’ bone marrow are mobilized and could contribute to both cell-tissue repair and anti-inflammatory response. This potential should be confirmed and further explored in larger studies where the characteristics of MSC from polytrauma patients would be compared at the genomic and functional levels with other critically ill patients, as well as the potential therapeutic role of MSC in this setting.

## Supplementary Information

**Additional file 1: Supplementary Table 1.** Clinical analytical values of polytraumatized patients upon hospital presentation.

**Additional file 2: Supplementary Figure 1.** Polytraumatized patients’ mean values during the in-hospital stay. Serum C-Reactive protein (A), lactate (B), procalcitonin (C), and absolute leucocyte count (D). Dotted lines are representing polynomic tendency lines (polynomial degree 4). Error bars are representing standard deviation (SD).

**Additional file 3: Supplementary Figure 2.** Polytraumatized patients’ scatter plots and linear correlations between severity scores and length of in-hospital stay. A. Injury severity score correlation with the whole in-hospital stay in days. *R2*=0.501. B. Injury severity score correlation with length of stay at the Intensive Care Unit in days. *R*^*2*^=0.566. C. New injury severity score correlation with length of stay at the Intensive Care Unit in days. *R*^*2*^=0.801.

**Additional file 4: Supplementary Figure 3.** NISS’ scatter plot and linear correlation to TNFα level of polytraumatized patients upon hospital admission (*R*^*2*^=0.439). NISS refers to new injury severity score.

**Additional file 5: Supplementary Figure 4.** Immunophenotypic characterization of MSCs. MSCs from polytraumatized patients (blue) and control group (orange) were compared to unstained cells (grey) used as a control for autofluorescence.

**Additional file 6: Supplementary Figure 5.** Fold increase in mRNA levels of pro-inflammatory, anti-inflammatory, and regulatory genes TNFα, TGFβ, IL-6, IL-10, PTGS2, IDO, and BDH2.

## Data Availability

The datasets supporting the conclusions of this article are available from the corresponding author upon reasonable request. Be aware that there are ethical and legal restrictions on sharing the original study datasets. The electronic health records data cannot be shared publicly because it consists of personal information from which it is difficult to guarantee de-identification (Law 03/2018 from Spanish Government - BOE-A-2018-16673).

## Abbreviations

MSCs: Mesenchymal stromal cells
ISS: Injury Severity Score
NISS: New Injury Severity Score
SIRS: Systemic inflammatory response syndrome
MOF: Multiorgan failure
DAMPs: Damage-associated molecular patterns
IL: Interleukin
ICU: Intensive care unit
LOS: Length of stay
MNCs: Mononuclear cells
McAb: Monoclonal antibodies
CFU-F: Fibroblast-like colony forming units
PD: Population doubling
CPD: Cumulative population doublings
