# Supplementary material for: MSCs from polytrauma patients: preliminary comparative study with MSCs from elective-surgery patients
Source: Stem Cell Res Ther. 2021 Aug 11;12:451. doi: 10.1186/s13287-021-02500-9 (PMC8356428; doi:10.1186/s13287-021-02500-9)
Supplement: Supplementary file 1 — Additional file 1: Supplementary Table 1. Clinical analytical values of polytraumatized patients upon hospital presentation. [file 13287_2021_2500_MOESM1_ESM.docx]

Supplementary Table 1. Clinical analytical values of polytraumatized patients upon hospital presentation.

|  | N | Minimum | Maximum | Mean | Standard deviation |
| --- | --- | --- | --- | --- | --- |
| Hemoglobin (g/dL) | 15 | 9.3 | 18.4 | **12.5** | 2.1 |
| Hematocrit (%) | 15 | 27.8 | 52.6 | **35.9** | 6.0 |
| Absolute Leukocyte count (x10^3^/µl) | 15 | 10.3 | 34.5 | **16.8** | 6.0 |
| Absolute platelet count (x10^3^/µl) | 15 | 127.0 | 318.0 | **208.0** | 46.1 |
| Glucose (mg/dL) | 16 | 119 | 442 | **205.9** | 87.8 |
| Urea (mg/dL) | 16 | 14.0 | 52.0 | **33.0** | 9.9 |
| Creatinine (mg/dL) | 16 | 0.56 | 1.14 | **0.81** | 0.2 |
| Ionic sodium (mmol/L) | 16 | 73 | 147 | **133.6** | 19.3 |
| Ionic Potassium (mmol/L) | 16 | 3.4 | 4.9 | **4.0** | 0.4 |
| Ionic chloride (mmol/L) | 16 | 101 | 114 | **106.3** | 3.6 |
| Prothrombin time – PT – (sec) | 16 | 12 | 112 | **84.4** | 23.0 |
| Activated partial thromboplastin time – APTT – (sec) | 16 | 24.0 | 33.6 | **27.7** | 2.6 |
| Fibrinogen (mg/dL) | 5 | 149 | 264 | **217.2** | 48.4 |
| INR | 16 | 0.90 | 1.24 | **1.09** | 0.09 |
| IL-6 (pg/mL) | 12 | 10.2 | 559.1 | **97.5** | 149.3 |
| TNFα (pg/mL) | 12 | 4.4 | 29.8 | **9.6** | 6.8 |
| C-Reactive protein – CRP – (mg/dL) | 14 | 0.6 | 13.6 | **6.3** | 4.8 |
| Erythrocyte sedimentation rate – ESR – (mm) | 12 | 4 | 43 | **21.1** | 11.4 |
| Procalcitonin (ng/mL) | 6 | 0.12 | 7.52 | **2.02** | 2.77 |
