# Supplementary figures and images for: MSCs from polytrauma patients: preliminary comparative study with MSCs from elective-surgery patients
Source: Stem Cell Res Ther. 2021 Aug 11;12:451. doi: 10.1186/s13287-021-02500-9 (PMC8356428; doi:10.1186/s13287-021-02500-9)

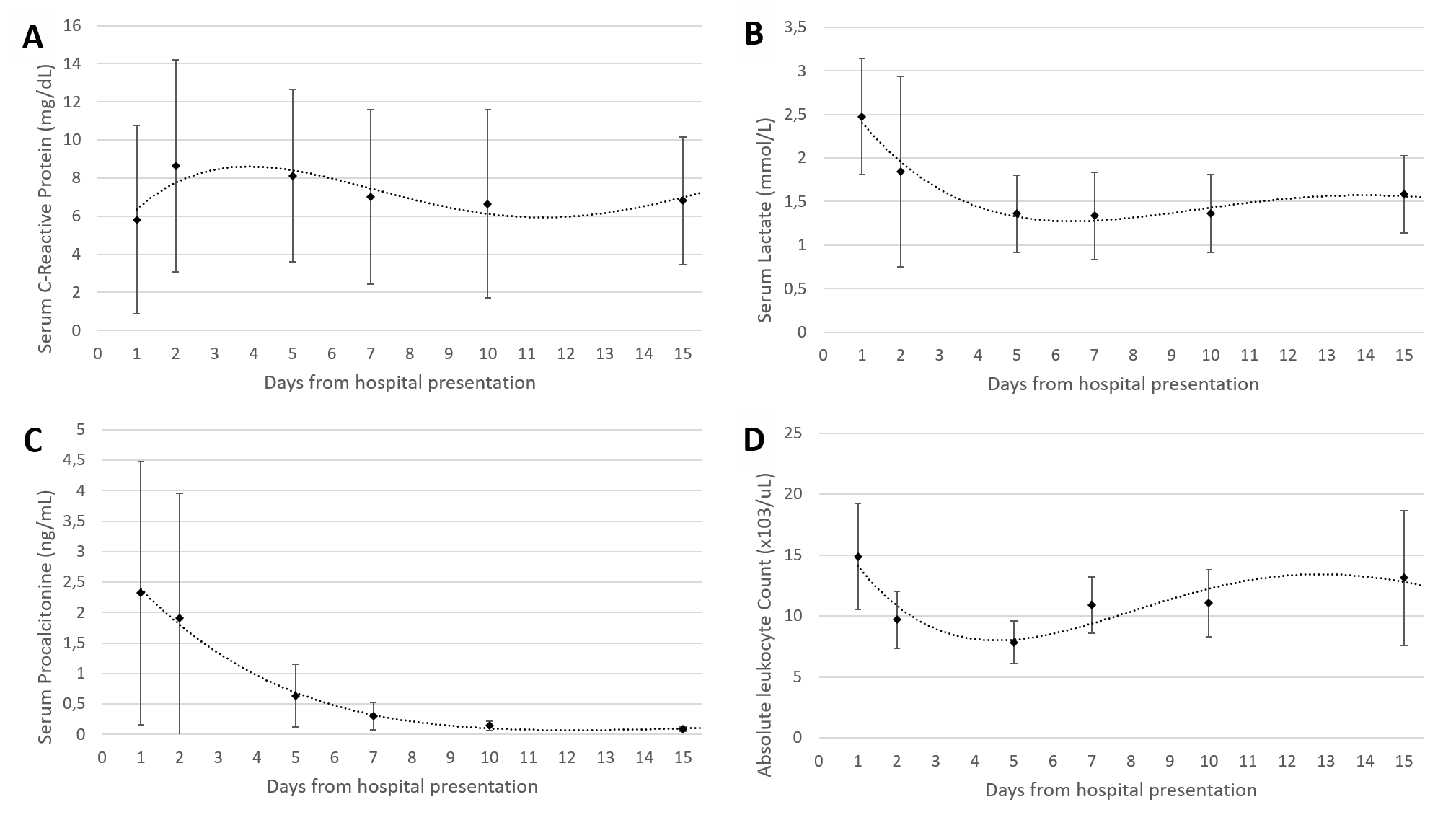

Supplement: Supplementary file 2 — Additional file 2: Supplementary Figure 1. Polytraumatized patients’ mean values during the in-hospital stay. Serum C-Reactive protein (A), lactate (B), procalcitonin (C), and absolute leucocyte count (D). Dotted lines are representing polynomic tendency lines (polynomial degree 4). Error bars are representing standard deviation (SD). [file 13287_2021_2500_MOESM2_ESM.tif]

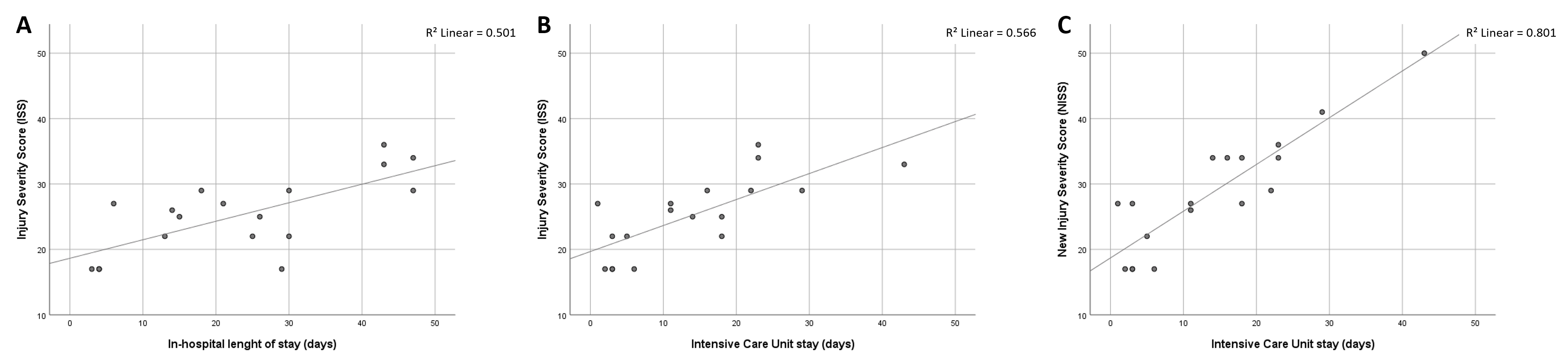

Supplement: Supplementary file 3 — Additional file 3: Supplementary Figure 2. Polytraumatized patients’ scatter plots and linear correlations between severity scores and length of in-hospital stay. A. Injury severity score correlation with the whole in-hospital stay in days. R2=0.501. B. Injury severity score correlation with length of stay at the Intensive Care Unit in days. R2=0.566. C. New injury severity score correlation with length of stay at the Intensive Care Unit in days. R2=0.801. [file 13287_2021_2500_MOESM3_ESM.tif]

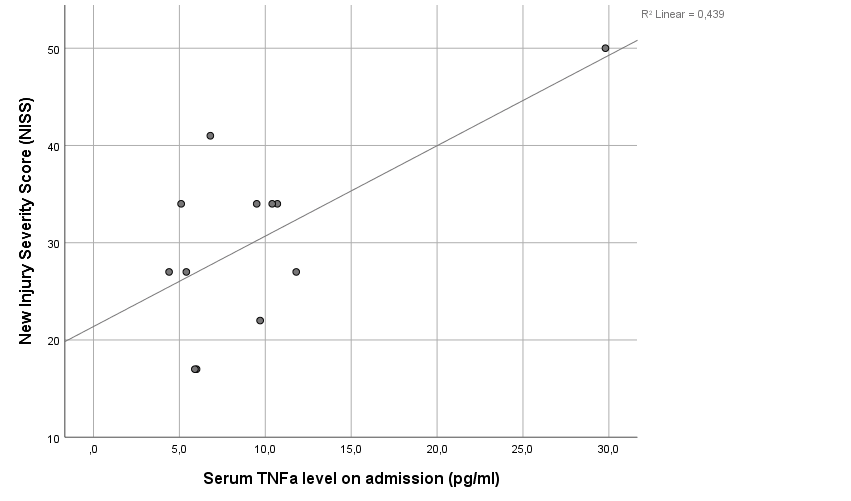

Supplement: Supplementary file 4 — Additional file 4: Supplementary Figure 3. NISS’ scatter plot and linear correlation to TNFα level of polytraumatized patients upon hospital admission (R2=0.439). NISS refers to new injury severity score. [file 13287_2021_2500_MOESM4_ESM.tif]

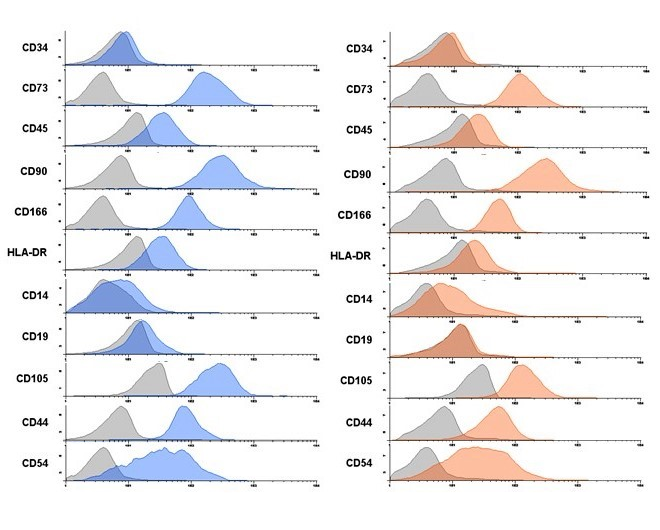

Supplement: Supplementary file 5 — Additional file 5: Supplementary Figure 4. Immunophenotypic characterization of MSCs. MSCs from polytraumatized patients (blue) and control group (orange) were compared to unstained cells (grey) used as a control for autofluorescence. [file 13287_2021_2500_MOESM5_ESM.tiff]

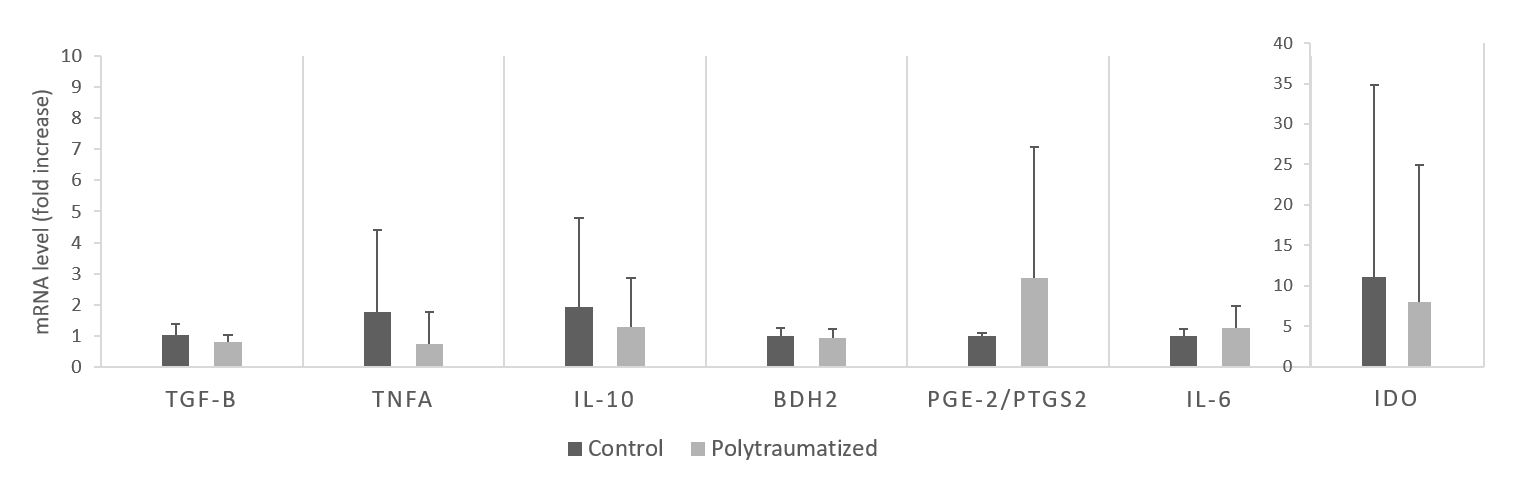

Supplement: Supplementary file 6 — Additional file 6: Supplementary Figure 5. Fold increase in mRNA levels of pro-inflammatory, anti-inflammatory, and regulatory genes TNFα, TGFβ, IL-6, IL-10, PTGS2, IDO, and BDH2. [file 13287_2021_2500_MOESM6_ESM.tif]
